# A Mixed-Methods Investigation of Facilitators to Accessing and Utilising Mental Health Services amongst Sri Lankan Australians

**DOI:** 10.3390/ijerph20075425

**Published:** 2023-04-06

**Authors:** Amanda Daluwatta, Kathryn Fletcher, Chris Ludlow, Ariane Virgona, Greg Murray

**Affiliations:** 1 Centre for Mental Health, School of Health Sciences, Swinburne University of Technology, Melbourne, VIC 3122, Australiagwm@swin.edu.au (G.M.); 2 Department of Psychological Sciences, School of Health Sciences, Swinburne University of Technology, Melbourne, VIC 3122, Australia; 3 Department of Psychology, Counselling and Therapy, School of Psychology and Public Health, La Trobe University, Melbourne, VIC 3086, Australia; avirgona@students.latrobe.edu.au

**Keywords:** facilitators, mental health service, enablers of care, Sri Lanka, Australia, mental health, help-seeking

## Abstract

Many individuals with mental health conditions avoid, delay, discontinue, or do not seek mental health services and treatments, despite the existence of evidence-based treatments and support methods. Little is known about the barriers to mental health service utilisation for Sri Lankan Australians, and there is no research on factors that facilitate access for this group. Using quantitative and qualitative methods, this study explored the perspectives of Sri Lankan Australians (*N* = 262) on the facilitators of mental health service utilisation. Participants rated a set of 18-items (Facilitator Set) and 7 public health interventions (Intervention Set) in relation to their capacity to improve the uptake of mental health services. Participants also completed two open-ended questions about the enablers to seeking professional mental health care. Descriptive statistics were used to summarise quantitative findings, while open-text responses were analysed using reflexive thematic analysis. The Facilitator Set demonstrated that trust in the provider and their confidentiality processes, positive relationships with mental health professionals, and the community having positive attitudes towards seeking help were the primary facilitators to seeking professional help. The Intervention Set suggested that raising community awareness of mental health conditions and providing public stigma reduction interventions may increase access to care. Themes identified in the open-ended responses included access to culturally safe and responsive services and clinicians, improved accessibility and affordability of services, trust, and a community-based approach to increasing mental health literacy and addressing stigma beliefs. Within its limitations, the present study’s findings suggest that providing culturally safe and responsive care, dispelling mental health stigma, and increasing knowledge of mental health conditions within Sri Lankan Australian communities are potential facilitating factors that would enable Sri Lankan Australians to seek and use mental health services. Implications for clinical care and future research are discussed.

## 1. Introduction

A “service gap” for mental health service use exists when individuals with mental conditions avoid, delay, discontinue, or do not use services or support at all, despite the existence of evidence-based treatment and support modalities [1]. Evidence demonstrates that Asian immigrant and refugee communities consistently use mental health services at significantly lower rates than the Australian-born population [2,3]. One Australian study found that the Sri Lankan community has one of the lowest mental health service access rates compared to the Australian-born population [4]. 

Identifying barriers and facilitators associated with mental health service use constitutes a fundamental initial step for understanding why there is a mental healthcare service gap for Asian and Sri Lankan migrants in Australia. Barriers are defined as the reasons or obstacles that prevent individuals from seeking, obtaining, or completing mental health treatment; whereas, facilitators are those factors that make the process of seeking, obtaining, or completing mental health treatment easier or more likely [5]. It could be argued that a clearer understanding of the enablers and facilitators to seeking mental health services could assist the field of clinical psychology to increase access to services, and help mental health professionals better serve the Sri Lankan Australian community [6].

Some literature has suggested that identifying as male [7], negative attitudes and beliefs towards mental illness in one’s family unit [8], and a lower ability to recognise depression among older Sri Lankan Australians [9] may act as barriers to professional help-seeking amongst Sri Lankan Australian populations. However, none of the extant literature has specifically investigated facilitators of mental health service use for Sri Lankan Australians. 

Although there is no data specifically focusing on facilitators for mental health care for Sri Lankan Australians, related data in culturally and linguistically diverse (CALD) immigrant women in Australia [10], African migrants in South Australia [11], and young people from refugee [12] and sub-Saharan African backgrounds [13] in Australia provide clues to factors that may also encourage Sri Lankan Australians to utilise mental healthcare facilities. Specifically, service-level cultural responsiveness [10], improving the affordability of care and transportation-related costs [11], perceived trustworthiness of help sources, and assurance that information disclosed would remain confidential [13] may also facilitate access among Sri Lankan Australians. Additionally, Australian research has found that matching clients from a non-English-speaking background with a bilingual, bicultural case manager resulted in a higher frequency and longer duration of contact with community mental health services than similar clients who were not matched with a bilingual case manager [14]. Therefore, access to bilingual, bicultural clinicians and to practitioners who prioritise developing a strong therapeutic relationship (especially the qualities of trust, understanding, respect, and a caring connection) and are sensitive to the client’s cultural background [12] may also influence Sri Lankan Australians’ use of mental health services. 

### The Present Study

The present study aimed to fill this gap in the literature by utilising a mixed-methods approach to identify perceived facilitators of Sri Lankan Australians seeking and using mental health services. Additionally, the present quantitative and qualitative investigation aimed to identify public health interventions that would improve the uptake of mental health services for a sample of Sri Lankan Australian adults. 

## 2. Method

### 2.1. Study Design and Procedure

As part of a broader cross-sectional survey of Sri Lankan Australians’ mental health and wellbeing, this exploratory study investigated facilitators of professional care for Sri Lankan Australians. As there were no existing quantitative measures on perceived facilitators to mental health service use available for migrant populations, the research team developed a set of items by employing a three-step methodology. First, an exhaustive literature review generated the initial 18-items. We chose to call this set of 18-items the “Facilitator Set” for ease of reference. The research team devised the root question: “*Out of the below factors, what would facilitate or enable you or a family member to seek and use mental health services?*”. In line with previous investigations into barriers to accessing care [15], the research team chose a four-point Likert scale with the response categories: “This would enable me to seek and use mental health services not at all (0)/a little (1)/quite a lot (2)/a lot (3)”. 

In step two, members of the research team with lived experience of being Sri Lankan Australian (*n* = 2) reviewed the draft Facilitator Set. The research team’s lived experience was incorporated into the set of items by slightly amending the content and adding an “other” option. In step three, a pilot study was conducted with Sri Lankan Australian community members (*n* = 9) for understandability and face validity. Based on participant feedback, the wording of some questions was revised for clarity and cultural relevance. 

The final version of the Facilitator Set was released in the “Sri Lankan Australians—Let’s Talk About Your Mental Health” cross-sectional, national survey that was delivered online via the Qualtrics survey platform. Data collection was between April 2020 and October 2020. Ethical approval was obtained from Swinburne University Human Research Ethics Committee (20202610-4168). All participants received written information about the study and provided consent before proceeding with the survey. Participants were informed that their identity would be anonymous, and that participation was voluntary. 

### 2.2. Participants and Recruitment

Individuals were eligible to participate if they were at least 18 years of age, English-speaking, self-identified as having Sri Lankan ancestry, and presently residing in Australia. For the purposes of this research study, “Sri Lankan Australians” were defined as individuals of Sri Lankan heritage who were currently living in Australia, regardless of their visa status. A purposive sampling strategy was employed to recruit participants, using a video advertisement that contained a hyperlink to the survey. Participants were recruited via community partners, clinicians and community leaders’ social media accounts, websites, radio station channels, and mailing lists. 

### 2.3. Measures

#### 2.3.1. Demographics

Age, gender, education, country of birth, ethnicity, religion, and number of years in Australia were examined.

#### 2.3.2. Facilitators to Seeking and Using Mental Health Services

Participants rated the degree to which the 18 treatment facilitators listed in the Facilitator Set were perceived to enable them to seek and use mental health services, with higher scores indicating a greater facilitator. Participants were given the opportunity to provide free-text responses to the following open-ended question at the end of the questionnaire: “*Please specify any other factors that would facilitate you or a family member in seeking a mental health service?*”. 

#### 2.3.3. Public Health Interventions

A list of seven public health interventions was also developed which sought to elicit participants’ input to provide service providers and mental health professionals with clear, actionable recommendations. We chose to call the 7-items the “Intervention Set”. The root question for the Intervention Set was: “*Rate each recommendation on how helpful it would be in improving uptake of mental health services, as well as Sri Lankan Australians’ mental wellbeing?*”. Participants rated the interventions on the following 4-point Likert scale: “This would help not at all (0)/a little (1)/quite a lot (2)/a lot (3)”, with higher scores representing greater facilitators to seeking treatment. At the end of the list, the following open-ended question was provided: “*Do you have any other recommendations?*”.

### 2.4. Statistical Analyses

#### 2.4.1. Quantitative Data

Data were analysed using IBM Statistical Package for the Social Sciences (SPSS) Version 28. As the Facilitator Set and Intervention Set were structured similarly to the Barriers to Access to Care Evaluation scale (BACE), the research team decided to also score these sets of questions in line with the BACE manual [15]. Descriptive statistics were used to summarise the pattern and distribution of responses for both the 18-item Facilitator Set and the 7-item Intervention Set. Specifically, percentages endorsing each facilitator as helpful to any degree and the mean of the response scores for each facilitator were calculated. 

#### 2.4.2. Qualitative Data

Participants were given two opportunities with slightly different prompts to speak about their perspectives of other facilitators to seeking and using mental health services. Initial exploration demonstrated that responses from the above open-ended questions could be meaningfully aggregated. The text responses were coded using QSR International’s nVivo 12 software and a reflexive thematic analysis method was applied to data extracts using principles guided by Braun and Clarke [16]. An inductive thematic analysis was chosen to identify patterned meaning across text responses [16]. Initially, the first author (AD) became familiar with the data by reading through the printed-out responses several times. The majority of coding was semantic; however, as each response was re-visited during the coding process, some latent coding was also applied to demonstrate broader concepts. Additionally, conversations with other authors were used to increase researcher reflexivity, and a reflective journal was used throughout the analysis process to record observations and personal reflections on how the first author’s own upbringing, ethnic background (Sri Lankan Australian), and relationship to the broader Sri Lankan Australian community impacted this research. Related codes were collated into potential meaningful themes which were then discussed with other authors. Mind maps were utilised to revise and refine themes and ensure themes were firmly grounded in the data. Unnecessary details were removed from the data extracts to aid readability and comprehension.

## 3. Results

### 3.1. Quantitative Findings

#### 3.1.1. Demographics

Table 1 shows the demographic characteristics of the final sample of 262 Sri Lankan Australians. The mean age of respondents was 31.7 years (*SD* = 11.5), and 71.8% of participants identified as female. The majority identified as Sinhalese (85.1%), followed by Tamil (5.7%). Most participants achieved an education level of an undergraduate degree or above.

#### 3.1.2. Facilitators to Seeking and Using Mental Health Services

**Figure 1:**
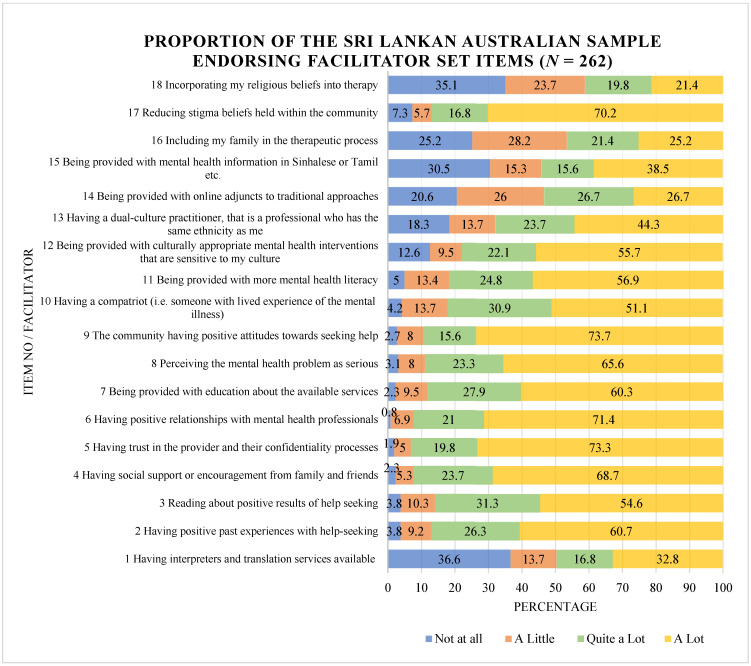
Proportion of the sample endorsing Facilitator Set items (*N* = 262).

Figure 1 provides a summary of the distribution of participant responses, expressed as a percentage out of 100%, for each item in the Facilitator Set. The stacked bar chart (Figure 1) indicates the extent to which participants endorsed each item within the Facilitator Set as enabling them to seek and use mental health services to any degree. For instance, 25.2% of participants reported that involving their family in the therapeutic process would ‘not at all’ enable them to seek and use mental health services. The mean scores for individual items within the Facilitator Set are included in Table S1 in the Supplementary Material.

The top three perceived facilitators based on mean ratings were “having trust in the provider and their confidentiality processes” (mean = 2.65), “having positive relationships with mental health professionals” (mean = 2.63) and “the community having positive attitudes towards seeking help” (mean = 2.60). In addition to these suggestions, having social support and encouragement from their social network (mean = 2.59), perceiving the mental health problem as serious (mean = 2.52), and reducing stigma beliefs held within the community (mean = 2.50) were also identified as having the potential to be helpful. As shown in Figure 1, the items that were most frequently recognised by participants as ‘not at all’ enabling access to services included “having interpreters and translation services available”, “incorporating my religious beliefs into therapy” and “being provided with mental health information in Sinhalese or Tamil etc.”.

#### 3.1.3. Public Health Interventions

**Figure 2:**
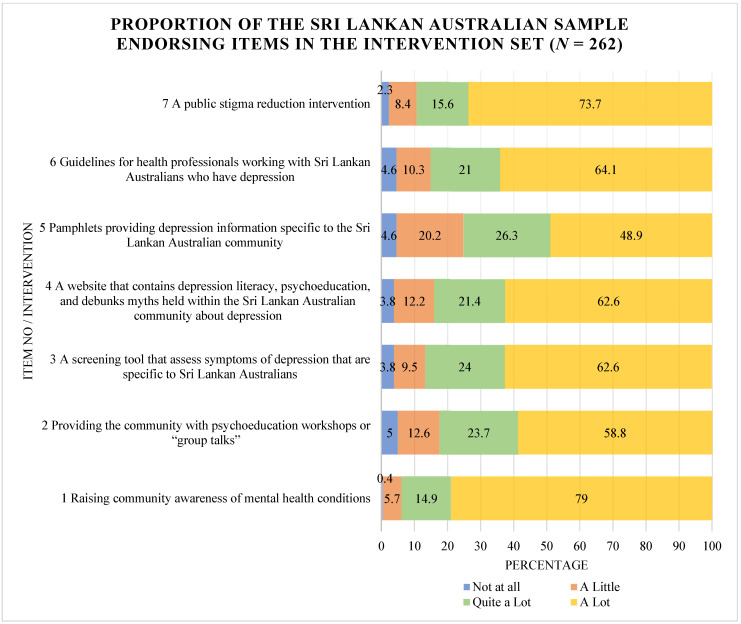
Proportion of the sample endorsing Intervention Set items (*N* = 262).

The frequency with which each intervention was believed to be helpful to any degree is shown in Figure 2. The mean scores for individual items within the Intervention Set are included in Table S2 in the Supplementary Material. The top four public health interventions believed to improve uptake of mental health services, as well as Sri Lankan Australians’ mental wellbeing (based on the mean), were “raising community awareness of mental health conditions” (mean = 2.73), “a public stigma reduction intervention” (mean = 2.61), “guidelines for health professionals working with Sri Lankan Australians who have depression” (mean = 2.45) and “a screening tool that assess symptoms of depression that are specific to Sri Lankan Australians” (mean = 2.45). 

### 3.2. Qualitative Findings

A total of *n* = 95 participants (36.3%) provided free-text answers to open-ended questions regarding facilitators to accessing care. Four overarching themes were identified: (1) a community-based approach to increasing mental health literacy and addressing stigma beliefs; (2) access to culturally safe services and culturally responsive clinicians; (3) trust; and (4) accessibility of services. Illustrative examples from the data are provided in-text for each theme. 

#### 3.2.1. A Community-Based Approach to Increasing Mental Health Literacy and Addressing Stigma Beliefs

Over half of the respondents suggested that encouraging discussion and open dialogue about mental wellbeing and illness and implementing measures to increase the mental health literacy of the Sri Lankan Australian community would likely facilitate access to and engagement with mental health services. Many participants provided suggestions on potential topics that could be covered as part of a mental health literacy intervention. For example, some participants stressed the importance of educating the community on “*how normal it is to be mentally unwell*”, “*debunking mental health myths*—i.e., *seeking help for illness will prevent you from getting a job, or taking medication will make you an addict*”, discussing how to maintain good mental health, and raising awareness on how to identify if there is a mental health problem (such as “*being able to understand what I am feeling and identify it as a mental illness or not. (i.e., is this just feeling sad or is this something more?)*”). Respondents additionally conveyed that mental health literacy interventions could include discussions around the aetiology of mental illness and how domestic violence, drug abuse, and exposure to traumatic events (including exposure to war) may precipitate mental health concerns. 

A large proportion of participants believed that addressing the stigma associated with mental health conditions within family and community circles was critical to improving Sri Lankan Australians access to services. One participant noted the importance of “*reducing stigma around mental health in the community through education and awareness. The Sri Lankan culture being collectivist like most Asian cultures, effective change around mental health should start at the community level rather than with the health system or the individual*”. Some participants noted how cultural beliefs about mental illness being a personal weakness or a lack of willpower resulted in public stigma and social judgement. One participant expressed how “*there is also the stigma that if you have been diagnosed with a mental illness like depression or anxiety, you are considered defective and many family/community members will look down upon you which escalates your condition. Breaking those societal expectations and understanding how it can come in many forms, from minor to severe and knowing how to identify and help others would help*”. 

Some of the participants suggested that incorporating lived/living experiences about “*positive mental health outcomes after accessing support including testimonials*”, as well as having “*young and old leaders*” and “*successful people who have come out the other end (or still struggling somewhat) speak about it*” would help reduce mental health-related stigma within the community. Additionally, participants recommended that the psychoeducation and stigma reduction campaigns be catered to issues specific to each age group (e.g., youth mental health vs. older mental health). For example, one respondent stated: “*I feel like a lot of the time our parents are quite dismissive of mental health problems because they think ‘well you grew up in a first world country, we provided everything for you, your life is so easy compared to ours what do you have to complain about’. So some education of breaking down the stigma in particular with youth mental health would be good*”.

Participants also suggested a variety of avenues that could be used to have community mental health discussions. “*Integrating mental health awareness strategies into popular Sri Lankan events* e.g., *Sri Lankan New Years Festival*”, utilising “*community hotspots (Temples, Churches etc.)*”, as well as utilising “*social media*”, “*Sri Lankan radio, news and television channels*” (especially for older Sri Lankan Australians) were proposed to increase mental health and illness awareness. Furthermore, few participants believed that providing physical resources such as “*pamphlets at the Temple*” or “*resources about how to talk to your family about mental illness*” and offering “*workshops or seminars*” would be beneficial. One participant also noted that “*speaking with Sri Lankan GPs and beginning at the grass root levels…beginning from places of worship to other community groups*” would improve the uptake of mental health services, as well as Sri Lankan Australians’ mental wellbeing. 

#### 3.2.2. Access to Culturally Responsive Clinicians and Culturally Safe Services

Most participants voiced that access to culturally safe, responsive care and mental health professionals would enable them and their community to seek mental health services. One participant noted that “*the most valuable thing would be to have practitioners who really understand cultural pressures and trauma. I believe that a lot of the mental stress in my family stems from the trauma they underwent during the war and subsequent separation from and fracturing of our extended family/community*”. This sentiment of desiring a professional who actively attempted to understand ones’ worldview and the socio-political and historical context of Sri Lanka, including the impact of the Civil War, displacement, and the ongoing ethnic and religious tensions in which that worldview developed, was a common theme among participants.

Beyond participants conveying the importance of practitioners having a broad understanding of their cultural background, numerous participants noted that knowing where to find and having access to bicultural clinicians and bilingual practitioners that provide therapy “*in languages that everyone can understand*” also played an important role in their inclination to seek help. Some participants described being in the dynamic process of constructing a multifaceted cultural identity during their acculturation to Australia; therefore, they commented wanting “*to talk to a Sri Lankan practitioner who also has experience growing up in a Westernised culture so they understand more of the difficulties of managing a dual identity*”. Participants also voiced wanting to be seen in their richness and complexity and requesting support navigating the multiple identities and worlds as well as intergenerational differences. For example, they explained “*we never really know who we are. In Australia we’re Sri Lankan but if we’re also out of place in Sri Lanka in many situations… The difference in the personal values is quite interesting*”; therefore, they noted needing *“people and culture you can relate to*”. Furthermore, participants noted that the intersectionality of two or more minority identity factors, such as being “*LGBTQIA+”* and being a Sri Lankan migrant, resulted in an identity which was often more complex due to the interaction of the individual components. Therefore, respondents suggested that the availability of more inclusive and intersectional services, where clinicians considered the impact of multiple identities (e.g., gender, ethnicity, and sexuality) would be helpful. 

Some participants suggested that community members may respond best to culturally adaptive and “*culturally specific interventions that may not be conventional but are still helpful*”. For example, one participant noted that having access to “*family therapy tailored to the modern Second Generation family, where there’s a cultural barrier between the children and parents*” and services that consider some family involvement would facilitate Sri Lankan Australians seeking mental health services. This multicultural counselling and therapy (MCT) characteristic of balancing the individualistic approach with a collectivistic reality that acknowledges client’s embeddedness in families, relationships with significant others, communities, and cultures [17] was explained by participants to be critical when working with clients from collectivist cultures, including Sri Lankan Australian community members. 

#### 3.2.3. Trust

A distrust of service providers and a fear of lack of confidentiality were seen as significant barriers to effective engagement with mental health services. Some participants were concerned that their use of mental health services would become known to others in their community. This was said to be because “*Confidentiality is very important. Not to gossip about it in the community*”. Therefore, defining and providing assurance of confidentiality was believed to be critical to establishing and maintaining trust. 

Additionally, some participants suggested that negative and unhelpful beliefs about psychology and mental health services were present in the community. One participant noted “*the stigma within the Sri Lankan community that people should turn to religion over science is (from my experience) at the heart of why Sri Lankans aren’t accessing the help they may need…So many people in the Sri Lankan community believe that psychology is just common sense that anyone could tell you about—‘why go to a psychologist when you can go to the temple and collect ‘pinna’ (karma points) by performing rituals’, or just think the problem through by yourself?*”. Therefore, this belief that “*psychology is waste of time, money, and reputation*”, and a general distrust in psychology being helpful in managing mental health concerns was also perceived to be impacting Sri Lankan Australians approaching professional help providers. 

#### 3.2.4. Accessibility of Services

Many participants reported the cost of care as the greatest factor impeding them from seeking mental health care. One participant noted “*I wish I could get the help if I can afford the cost. Just like how I am feeling now, I know I need help. But I can’t get that help. So all I have in my hand is to figure out things by my own. And unfortunately, this is the case for many of my friends*”. This barrier was most apparent for community members who did not have citizenship such as international students or “*temporary residents* [who] *do not get free health care services*”.

Once the cost barrier was resolved, new issues arose, including the availability of timely care and a lack of continuity of care. Participants noted that “*knowing that there will be ongoing culturally appropriate care in the community (after an acute admission/outpatient clinic)*” and having access to professional support in “*urgent situations*” would greatly assist them in accessing services. 

## 4. Discussion

To our knowledge, the present study is the first to specifically investigate factors that may facilitate Sri Lankan Australians in accessing and utilising mental health services. Responses to the Facilitator Set suggested that having trust in the provider and their confidentiality processes would most likely assist Sri Lankan migrants in Australia in accessing and utilising mental health services; whereas responses to the Intervention Set demonstrated that raising community awareness of mental health conditions and implementing a public stigma reduction intervention were important facilitating factors. The qualitative findings complemented findings from the Facilitator and Intervention sets by confirming that establishing trust, increasing mental health literacy, and addressing stigma beliefs may facilitate treatment-seeking amongst the Sri Lankan Australian population. Additionally, the qualitative findings suggested that improving access to culturally safe services and culturally responsive clinicians was vital to improving access to care. 

Taken together, the quantitative and qualitative evidence can be grouped into three sections regarding addressing practical barriers including trust in the providers’ confidentiality processes; accessibility of culturally safe and responsive mental health care; and community-based interventions aimed at increasing mental health literacy and addressing public stigma. This study was also extremely successful in using quantitative and qualitative methods to identify a series of findings that translated into recommendations that can assist clinicians and service providers to better address Sri Lankan Australians’ mental health needs. 

### 4.1. Access to Culturally Safe and Responsive Services and Clinicians

Participants believed that the availability of more culturally safe, responsive, and aware clinical environments that allowed them to disclose their life narratives would improve uptake of mental health services. In the present study, findings from the Facilitator Set demonstrated that 55.7% of respondents noted that being provided with culturally appropriate mental health interventions that were sensitive to their culture would enable them to seek and use mental health services ‘a lot’. Participants’ suggestions on how to improve the cultural safety and responsiveness of Australian mental health care services included both suggestions for individual practitioners and broader service-level change. The qualitative findings noted that practitioners’ lack of awareness of premigration contexts, such as the socio-political and historical context of Sri Lanka, including cultural pressures and experiences of cumulative trauma, and postmigration experiences, such as challenges of having a bicultural identity, led to concerns that their problems would not be understood or taken seriously by practitioners. Therefore, in this context, cultural safety requires cultural understanding, because clients cannot feel safe accessing services if they do not feel their cultures, as well as intersectional and bicultural identities, are understood and respected [18]. 

As shown in Figure 1, on a service level, some strategies to overcome language barriers such as having access to appropriate interpreter services and being provided with mental health information in Sinhala or Tamil were not highly endorsed as enabling access to services. However, qualitative findings demonstrated that having access to bilingual practitioners was believed to be helpful in addressing communication and language barriers. Beyond the language barrier, qualitative findings suggested that knowing where to find and having access to bicultural clinicians as part of service provision would encourage Sri Lankan Australians to access care. Interestingly, having a dual-culture practitioner, that is, a professional who had the same ethnicity as the respondents, was also endorsed as being ‘a lot’ helpful in facilitating or enabling the use of mental health services for 44.3% of respondents. These findings align with recently recommended strategies to increase the acceptability of services among African communities in Australia; where service providers with African background and understanding of the past experiences of African migrants was considered to be important to increase access to mental health services among African migrants [19]. 

We propose that service providers try to implement measures which improve the cultural safety and responsiveness of mental healthcare services. This approach would encourage an individualised and holistic mental health care plan, which took into consideration all the multifactorial influences (acculturation, socioeconomic status, gender, privilege, ethnic heritage, etc.) impacting on the client’s reality [10]. For example, considering the impact of collectivist cultures on the mental health consultation by providing access to family therapy or having some level of family involvement may lead to higher utilisation rates. 

### 4.2. Addressing Practical Barriers: Confidentiality, Trust, and Affordability

As well as ensuring the cultural responsiveness of mental health clinicians, at a service provider level, changes in the affordability of mental health care and providing assurance of confidentiality were perceived as most likely to improve the uptake of mental health help-seeking for Sri Lankan Australians. 

Both the Facilitator Set and qualitative findings illustrated that a major concern for many of the study’s participants was confidentiality and trust in mental health services and clinicians. In fact, 73.3% of participants noted that having trust in the provider and their confidentiality processes would enable them to seek and use mental health services ‘a lot’. One participant noted that “*having not just a translator, but a trusted professional who can actually speak the language, and engage directly with the client would make a world of difference. This person must be trusted to keep services confidential and should conduct their practice in a way that community members shouldn’t fear that they’d be spotted by someone else in the community*”. This participant’s response suggests that concerns about confidentiality may also be related to stigma, where a fear of a breach in confidentiality stems from the fear of stigma and embarrassment, should other community members find out that they had sought help [20]. This concern was also identified in a study of experienced clinicians, who suggested that fears about loss of confidentiality and information being leaked to other community members, colleagues, and even future in-laws was a barrier to mental health treatment among South Asians living in the United States [21]. To address mistrust and to assist in establishing therapeutic rapport, clinicians could explicitly state a commitment to confidentiality, as well as explain the purpose of gathering information, how the information will be used, circumstances where they may have to break confidentiality such as acute risk, and how they would proceed in such an event [22]. 

The cost of professional care was another factor perceived to be impacting Sri Lankan Australians seeking mental health care. The unaffordability of healthcare services was also identified as a barrier to accessing mental health services among CALD populations in three regional towns in South Australia [23] and African migrants in South Australia [11]. Therefore, an investment in mental healthcare [24,25], including an increase in federal and state funding for mental health services tailored to CALD populations, including Sri Lankan migrants in Australia, as well as a widespread extension of bulk billing [26] may assist in improving the affordability of professional services. 

### 4.3. A Community-Based Approach to Increasing Mental Health Literacy and Addressing Public Stigma Beliefs

Findings from the Intervention Set and qualitative exploration suggested that disseminating tailored mental health literacy, with an emphasis on explaining how to recognise different mental health conditions and symptoms was essential to enabling professional care for Sri Lankan Australians. Participants noted that a community-based approach that utilised social media and Sri Lankan radio and television to have discussions that demystified and normalised experiencing periods of mental illness may assist in fostering positive attitudes towards mental health. Raising awareness about mental health issues and how to navigate mental health services through online resources, printed resources and community-based seminars and workshops has also been a suggested strategy for improving access to mental health care for Farsi-speaking immigrants in Quebec [18]. 

In addition to poor mental health literacy, mental health stigma and shame also appeared to influence access to mental health services among Sri Lankan migrants in Australia. Stigma has been described as having two components: public stigma (a set of negative attitudes and beliefs that motivate individuals to fear, reject, avoid, and discriminate against people with mental illness) [27] and self-stigma (when a person with a mental illness internalises the public stigma and experiences diminished self-esteem and self-efficacy) [28]. When the public and community attach a stereotype to individuals with mental illness and proceed to discriminate against them, those who believe they may have a mental illness tend to shield themselves from this discrimination by hiding their illness and avoiding visiting mental health services [29]. Research with African migrants in South Australia has demonstrated that mental health stigma has a negative impact on participants’ social life and relationships; for example, some participants noted that community members with mental health problems were regarded as outcasts, as well as negatively judged, isolated, and labelled [11]. Additionally, this mental health stigma from other community members appeared to deter African migrants from accessing mental health services in South Australia [11]. 

In the current study, fear of negative social consequences and discrimination associated with public stigma also appears to deter Sri Lankan Australian community members from engaging with mental health services. In order to combat stigma, the present study’s participants suggested educating and providing information to the community as well as sharing stories of community leaders and members who have experienced periods of mental illness to help debunk myths held within the community. These recommendations from respondents were similar to a study conducted with American university students with Indian and Pakistani ancestry, who also suggested that working to unpack mental health stigma from within the community and create productive conversations would likely yield a better understanding for South Asian individuals of their own mental health, as well as a greater awareness and capacity for helping others [6].

### 4.4. Limitations

Despite these interesting findings, the study had several limitations. First, although all respondents were provided with the option to respond to open-text questions regarding facilitators to accessing mental health care, the depth of the reflexive thematic analysis of open-ended responses was limited as we were unable to ask follow-up questions or probe for further detail. As such, findings from the present study should be considered as a preliminary step towards better characterising facilitators of mental health service use. Future qualitative research using semi-structured interviews or focus-group formats may help expand upon the findings identified in this study. Second, as explained in other explorations which utilised the same cross-sectional survey [9], the disproportionate underrepresentation of Tamil participants and the overrepresentation of Sinhalese participants were significant weaknesses of the current study and limit the interpretation and generalisation of the results. Third, this study adopted an exploratory approach to identify a range of factors that may promote help-seeking behaviours for mental health in the Sri Lankan Australian community. Future research studies can expand on the present investigation by conducting multivariate analyses using sufficient sample sizes to explore the influence of individual attributes, such as age and educational attainment, on the perceived facilitators identified in this study. Finally, participants were provided the Facilitator Set and Intervention Set of potential enabling factors before the open-text questions were presented; therefore, it is possible that participants’ qualitative responses were influenced by the quantitative options. Nevertheless, these findings based on the first Sri Lankan Australian community sample provide much-needed insight into factors that may assist in uptake of care and point to targets for further research and tailored mental health promotion efforts.

## 5. Conclusions

The present study identified a range of potential enabling factors to mental health service utilisation among Sri Lankan Australian respondents. Having trust in the provider and their confidentiality processes, access to affordable services, having positive relationships with mental health professionals, and the community having positive attitudes towards seeking help were suggested facilitators to accessing mental health services among Sri Lankan migrant communities in Australia. Pending the replication of the present findings using different methods in other samples, the findings also suggested several ways forward. First, a community-based approach designed to improve Sri Lankan Australians’ understanding and recognition of their own symptoms and conditions, as well as a reduction of the public stigma associated with mental illness and mental health help-seeking, may assist in enabling access to mental health services. Second, improving mental health service provision by providing culturally safe and responsive services and access to bicultural clinicians could be another factor that would improve the accessibility of mental health services for the Sri Lankan Australian community. Therefore, when services become community-based, culturally safe, and trustworthy, more migrants may access care before their symptoms become severe and their problems affect their social functioning [18].

## Figures and Tables

**Table 1:** Demographics and other characteristics of the sample (*N* = 262).

Variables	Frequency	%
**Gender**		
Male	74	28.2
Female	188	71.8
**Age group (*Mean* = 31.7; *SD* = 11.5)**		
18–29 years	159	60.7
30–39 years	53	20.2
40–49 years	24	9.2
50–59 years	17	6.5
60–69 years	8	3.0
70 and over	1	0.4
**Highest level of education**		
Secondary school or equivalent	34	13.0
TAFE course or equivalent	20	7.6
Undergraduate degree	118	45.0
Postgraduate degree	90	34.4
**Country of birth**		
Australia	86	32.8
Sri Lanka	161	61.5
Other	15	5.7
**Years in Australia (*Mean* = 19.1; *SD* = 9.8)**		
Under 10 years	61	23.3
11 to 20 years	59	22.5
21 to 30 years	122	46.6
Above 31 years	20	7.6
**Ethnicity**		
Sinhalese	223	85.1
Tamil	15	5.7
Other	24	9.2
**Religion**		
Buddhist	158	60.3
Christian	16	6.1
Roman Catholic	31	11.8
Atheist or Agnostic	34	13.0
Other	23	8.8

*Note*: Standard Deviation (*SD*).

## Data Availability

The dataset presented in this article is not publicly available, but the dataset is available from the senior author on reasonable request.

## Supplementary Materials

The following supporting information can be downloaded at: https://www.mdpi.com/article/10.3390/ijerph20075425/s1, Table S1: The proportion of the Sri Lankan Australian sample endorsing Facilitator Set items and the mean score for each item (*N* = 262), Table S2: The proportion of the Sri Lankan Australian sample endorsing Intervention Set items and the mean score for each item (*N* = 262).
