# Supplementary material for: A Mixed-Methods Investigation of Facilitators to Accessing and Utilising Mental Health Services amongst Sri Lankan Australians
Source: Int J Environ Res Public Health. 2023 Apr 6;20(7):5425. doi: 10.3390/ijerph20075425 (PMC10093901; doi:10.3390/ijerph20075425)
Supplement: Supplementary file 1 [file ijerph-20-05425-s001.zip › ijerph-2276522-supplementary.pdf]

A Mixed-Methods Investigation of Facilitators to Accessing and Utilising Mental Health Services amongst Sri Lankan Australians

Amanda Daluwatta, Kathryn Fletcher, Chris Ludlow, Ariane Virgona & Greg Murray

Supplementary material

Table S1. The proportion of the Sri Lankan Australian sample endorsing Facilitator Set items and the mean score for each item (*N* = 262)

	% (n)				
Item number/ Facilitator	Not at all	A Little	Quite a Lot	A Lot	Mean (Std. Deviation)
**1** Having interpreters and translation services available	36.6 (96)	13.7 (36)	16.8 (44)	32.8 (86)	1.46 (1.28)
**2** Having positive past experiences with help-seeking	3.8 (10)	9.2 (24)	26.3 (69)	60.7 (159)	2.44 (0.81)
**3** Reading about positive results of help-seeking	3.8 (10)	10.3 (27)	31.3 (82)	54.6 (143)	2.37 (0.82)
**4** Having social support or encouragement from family and friends	2.3 (6)	5.3 (14)	23.7 (62)	68.7 (180)	2.59 (0.70)
**5** Having trust in the provider and their confidentiality processes	1.9 (5)	5.0 (13)	19.8 (52)	73.3 (192)	2.65 (0.67)
**6** Having positive relationships with mental health professionals	0.8 (2)	6.9 (18)	21.0 (55)	71.4 (187)	2.63 (0.65)
**7** Being provided with education about the available services	2.3 (6)	9.5 (25)	27.9 (73)	60.3 (158)	2.46 (0.76)
**8** Perceiving the mental health problem as serious	3.1 (8)	8.0 (21)	23.3 (61)	65.6 (172)	2.52 (0.77)
**9** The community having positive attitudes towards seeking help	2.7 (7)	8.0 (21)	15.6 (41)	73.7 (193)	2.60 (0.75)
**10** Having a compatriot (i.e. someone with lived experience of the mental illness)	4.2 (11)	13.7 (36)	30.9 (81)	51.1 (134)	2.29 (0.86)
**11** Being provided with more mental health literacy	5.0 (13)	13.4 (35)	24.8 (65)	56.9 (149)	2.34 (0.89)
**12** Being provided with culturally appropriate mental health interventions that are sensitive to my culture	12.6 (33)	9.5 (25)	22.1 (58)	55.7 (146)	2.21 (1.06)
**13** Having a dual-culture practitioner, that is a professional who has the same ethnicity as me	18.3 (48)	13.7 (36)	23.7 (62)	44.3 (116)	1.94 (1.15)
**14** Being provided with online adjuncts to traditional approaches	20.6 (54)	26.0 (68)	26.7 (70)	26.7 (70)	1.60 (1.09)
**15** Being provided with mental health information in Sinhalese or Tamil etc.	30.5 (80)	15.3 (40)	15.6 (41)	38.5 (101)	1.62 (1.27)
**16** Including my family in the therapeutic process	25.2 (66)	28.2 (74)	21.4 (56)	25.2 (66)	1.47 (1.12)
**17** Reducing stigma beliefs held within the community	7.3 (19)	5.7 (15)	16.8 (44)	70.2 (184)	2.50 (0.90)
**18** Incorporating my religious beliefs into therapy	35.1 (92)	23.7 (62)	19.8 (52)	21.4 (56)	1.27 (1.16)

Note: 'Not at all' = 0; 'a little' = 1; 'quite a lot' = 2; 'a lot' = 3

Table S2. The proportion of the Sri Lankan Australian sample endorsing Intervention Set items and the mean score for each item (*N* = 262)

	% (n)				
Item number/ Recommendation	Not at all	A Little	Quite a Lot	A Lot	Mean (Std. Deviation)
**1** Raising community awareness of mental health conditions	0.4 (1)	5.7 (15)	14.9 (39)	79.0 (207)	2.73 (0.58)
**2** Providing the community with psychoeducation workshops or "group talks"	5.0 (13)	12.6 (33)	23.7 (62)	58.8 (154)	2.36 (0.89)
**3** A screening tool that assess symptoms of depression that are specific to Sri Lankan Australians	3.8 (10)	9.5 (25)	24.0 (63)	62.6 (164)	2.45 (0.82)
**4** A website that contains depression literacy, psychoeducation, and debunks myths held within the Sri Lankan Australian community about depression	3.8 (10)	12.2 (32)	21.4 (56)	62.6 (164)	2.43 (0.85)
**5** Pamphlets providing depression information specific to the Sri Lankan Australian community	4.6 (12)	20.2 (53)	26.3 (69)	48.9 (128)	2.19 (0.92)
**6** Guidelines for health professionals working with Sri Lankan Australians who have depression	4.6 (12)	10.3 (27)	21.0 (55)	64.1 (168)	2.45 (0.86)
**7** A public stigma reduction intervention	2.3 (6)	8.4 (22)	15.6 (41)	73.7 (193)	2.61 (0.74)

Note: 'Not at all' = 0; 'a little' = 1; 'quite a lot' = 2; 'a lot' = 3
